# Characterization and Biofungicide Potential of a Novel Antifungal defensin, K4CBP6, from *Solanum lycopersicum* L.

**DOI:** 10.1007/s12602-025-10865-z

**Published:** 2025-12-08

**Authors:** Rebeka Papp, Péter Poór, Zalán Czékus, Györgyi Váradi, Zoltán Kele, Attila Borics, Gábor Bende, Kata Horváti, Gábor K. Tóth, László Galgóczy, Liliána Tóth

**Affiliations:** 1 Department of Biotechnology and Microbiology, Faculty of Science and Informatics, University of Szeged, Szeged, Hungary; 2 Doctoral School of Biology, Faculty of Science and Informatics, University of Szeged, Szeged, Hungary; 3 Department of Plant Biology, Faculty of Science and Informatics, University of Szeged, Szeged, Hungary; 4 Department of Medical Chemistry, Albert Szent-Györgyi Medical School, University of Szeged, Szeged, Hungary; 5 Institute of Biochemistry, HUN-REN Biological Research Centre, Szeged, Hungary; 6 Department of Theoretical Health Sciences and Health Management, Faculty of Health Sciences and Social Studies, University of Szeged, Szeged, Hungary; 7 Institute of Materials and Environmental Chemistry, HUN-REN Research Centre for Natural Sciences, Budapest, Hungary; 8 MTA-SZTE Biomimetic Systems Research Group, University of Szeged, Szeged, Hungary

**Keywords:** γ-core peptide, Antifungal defensin, Biofungicide, Plant pathogenic fungus, Plant protection, *Solanum lycopersicum* L.

## Abstract

The global rise in resistance to chemical fungicides and their strict regulation by the EU, has created an urgent need for alternative antifungal strategies in agriculture. Plant defensins represent promising alternatives owing to their broad-spectrum antifungal activity, structural stability, and low toxicity to mammalian cells and plants. In this study, we identified and characterized a novel antifungal defensin, K4CBP6, from *Solanum lycopersicum* L., along with its γ-core peptide derivatives, K4CBP6γ1 and K4CBP6γ2, as potential biofungicide agents. Protein database mining revealed a widespread distribution of K4CBP6 homologs within the Solanaceae family. Recombinant K4CBP6 (rK4CBP6) was successfully produced using a *Komagataella phaffii*-based expression system, while K4CBP6γ1 and K4CBP6γ2 were chemically synthesized. Structural analyses via electrospray ionization mass spectrometry and electronic circular dichroism spectroscopy confirmed a cysteine-stabilized α-helix β-strand folded structure for rK4CBP6. In vitro susceptibility assays demonstrated that both rK4CBP6 and K4CBP6γ2 exhibited antifungal activity against major tomato pathogens, *Botrytis cinerea*, *Cladosporium herbarum*, and *Fusarium oxysporum* with minimum inhibitory concentrations ranging from 12.5 to 25 µg ml^-1^. Furthermore, neither rK4CBP6 nor K4CBP6γ2 exhibited cytotoxic effects on mammalian cell lines or adverse effects in animal and plant model systems even at concentrations of 200 and 400 µg ml^-1^. Proof-of-concept experiments on tomato plants and fruits confirmed their protective efficacy against *B. cinerea* and *C. herbarum*. These findings highlight the potential of rK4CBP6 and K4CBP6γ2 as sustainable biofungicide candidates for plant disease management, owing to their antifungal efficacy both in vitro and in planta, along with their lack of cytotoxic effects.

**Supplementary Information:**

The online version contains supplementary material available at 10.1007/s12602-025-10865-z.

## Introduction

Crop pests and diseases contribute to annual crop yield losses ranging from 20% to 40% [1]. Microbial pathogens, particularly phytopathogenic fungi, are responsible for approximately 80% of all plant diseases, leading to substantial economic and food supply losses worldwide [2]. Beyond their direct impact on yield, certain fungal species produce mycotoxins that contaminate food and feed, posing serious health risks to both humans and animals [3]. Environmental factors further aggravate fungal proliferation. Climate change and rising temperatures promote the spread and reproduction of pathogenic fungi, even in previously unaffected regions [4]. Moreover, monoculture cultivation provides favorable conditions for fungal persistence and accelerates the evolution of hypervirulent and fungicide-resistant strains [5]. For decades, the predominant strategy to combat fungal diseases in agriculture has been the use of chemical fungicides due to their accessibility, cost-effectiveness, and broad availability in diverse formulations [6]. However, their intensive and often indiscriminate application has led to several drawbacks, including environmental pollution, biodiversity loss, and risks to human and animal health [7]. Furthermore, frequent, and improper fungicide application contributes to the development of resistance or tolerance [8]. Consequently, several countries have introduced stricter regulations and have banned or restricted the use of hazardous synthetic fungicides [9]. These limitations have driven the search for novel, environmentally friendly, and sustainable alternatives for fungal disease management.

Biocontrol strategies have emerged as promising and sustainable approaches to plant diseases management [10, 11]. These mechanisms involve the use of beneficial microorganisms or their metabolites to inhibit plant pathogens either directly, through antagonism and competition, or indirectly, by eliciting plant immune responses [12–14]. Among the molecules identified as potential biofungicides, antimicrobial peptides (AMPs) have garnered considerable attention owing to their potent and broad-spectrum antifungal activity, which continues to fuel growing scientific interest. AMPs are small, cationic peptides produced by a wide range of organisms, including plants, animals, and fungi, as part of their innate immune defense and exhibit a broad-spectrum antimicrobial activity [15, 16].

Among AMPs, defensins represent one of the largest and most extensively studied groups. Plant defensins are small (45–54 amino acids, 5–7 kDa), positively charged, amphipathic peptides that exhibit broad-spectrum antimicrobial activity, particularly strong antifungal properties [16–19]. Despite their diverse amino acid sequences, plant defensins share a highly conserved tertiary structure, comprising an α-helix and three antiparallel β-sheets arranged in a βαββ fold [20–22]. Their structural stability is reinforced by four intramolecular disulfide bridges between eight cysteine residues in a defined bonding pattern, which confers resistance to extreme environmental conditions such as high temperature, wide pH range, and proteolytic degradation [23]. A key determinant of their antifungal mechanism is the evolutionarily conserved γ-core motif (GXCX₃₋₉C, where X represents any amino acid). Structurally, this motif adopts a conserved three-dimensional β-hairpin conformation composed of two antiparallel β-strands connected by a flexible loop, contributing to its characteristic amphipathic and cationic nature [24]. The positively charged residues facilitate binding to the negatively charged plasma membrane of fungal pathogens [18, 25, 26]. Based on their mode of action, plant defensins are classified into two groups: morphogenetic defensins inhibiting fungal hyphal growth by inducing branching, and nonmorphogenetic defensins disrupting the fungal cell membrane [21, 22, 27]. Several studies have already reported the antifungal activity of synthetic peptides derived from the γ-core regions of plant defensins [18, 24, 27, 28]. In addition to their structural and functional advantages, the γ-core regions of plant defensins offer a practical benefit from an application standpoint. Their short length enables cost-effective chemical synthesis compared to full-length defensins, facilitating large-scale production and structural optimization for agricultural use. This makes γ-core peptides attractive candidates for developing sustainable and economically viable peptide-based biofungicides [25].

Plant defensins are promising alternatives to chemical fungicides, as they combine several desirable attributes, including broad antifungal specificity, environmental stability, low cytotoxicity to plants, and mammalian cells, and minimal risk of resistance development [16, 21, 29, 30]. Their potential applications include genetic engineering of defensin-expressing transgenic crops, which have shown enhanced resistance to a wide range of fungal pathogens [21, 26, 31]. However, the integration of genetically modified plants into agriculture remains challenging due to strict regulatory frameworks and widespread consumer opposition [32, 33]. An alternative approach is the direct use of defensin-based biofungicides. However, several limitations hinder their practical use, such as limited natural availability, high production costs, incomplete knowledge of their full antifungal spectrum, potential long-term effects on plants and humans [25, 31].

Despite the challenges, plant defensins represent a promising and environmentally sustainable solution for fungal pathogen control in agriculture. However, their application requires further research and development to optimize their effectiveness in crop protection. Previously, Rigano et al. [34] identified proteins with putative antimicrobial activity in *Solanum lycopersicum* L. Among these, the DEFL1 (Solyc07g007760) was highlighted as a candidate antifungal defensin [34–36]. To date, this protein has neither been isolated nor functionally characterized. Notably, defensins are referenced under various nomenclatures across different databases. At the outset of our research, this protein was listed in the UniProt database as K4CBP6 (UniProt ID: K4CBP6_SOLLC), and thus, this identifier was adopted in our study.

In this study, we characterized this plant defensin and investigated its biofungicidal potential in plant protection. To assess it, recombinant K4CBP6 (rK4CBP6) was produced, using a *Komagataella phaffii* (formerly *Pichia pastoris*)-based expression system, and its γ-core peptide derivatives were chemically synthesized. Antifungal susceptibility and toxicity assays were performed, and proof-of-concept experiments on tomato plants confirmed the protective efficacy of both rK4CBP6 and its γ-core peptide, supporting their potential as sustainable biofungicides.

## Materials and methods

### Strains and Media

The heterologous expression of K4CBP6 was conducted in *K. phaffii* KM71H (arg4 aox1::ARG4), which was maintained on yeast extract peptone dextrose (YDP) agar plates containing 1% yeast extract, 2% peptone, 2% dextrose, and 2% agar (w/v) at 4 °C. The fungal strains involved in susceptibility experiments are listed in Table 1. These plant pathogenic fungi were maintained on potato dextrose agar (PDA, Sigma-Aldrich, St. Louis, MO, USA) at 4 °C. Antifungal susceptibility tests were performed in ten-fold diluted potato dextrose broth (0.1× PDB, Sigma-Aldrich, St. Louis, MO, USA) to assess the efficacy of rK4CBP6 and γ-core peptide derivatives against plant pathogenic fungi.

**Table 1:** In vitro minimum inhibitory concentrations (MICs, µg ml^− 1^) of recombinant K4CBP6 (rK4CBP6) and its γ-core peptide derivatives (K4CBP6γ1 and K4CBP6γ2) against plant pathogenic fungi in 0.1 × potato dextrose broth after incubation at 24 °C for 72 h

Filamentous fungi (origin of isolate)	rK4CBP6	K4CBP6γ1	K4CBP6γ2
*Aspergillus flavus* SZMC 20745 (Corn/Hungary)	> 200	n.d.	n.d.
*Aspergillus niger* SZMC 0145 (Fruits/Hungary)	> 200	n.d.	n.d.
*Botrytis cinerea* SZMC 21472 (*Rubus idaeus*/Hungary)	**25**	> 400	**25**
*Cladosporium herbarum* FSU 1148 (n.d.)	**25**	**400**	**12.5**
*Fusarium avenaceum* SZMC 11044 (n.d.)	**50**	n.d.	n.d.
*Fusarium cerealis* SZMC 11045 (n.d.)	**25**	n.d.	n.d.
*Fusarium culmorum* SZMC 11039 (Vegetables)	**25**	n.d.	n.d.
*Fusarium graminearum* SZMC 11032 (Corn/South Africa)	**25**	n.d.	n.d.
*Fusarium subglutinans* CBS 747.97 (Zea mays/USA)	> 200	> 400	**25**
*Fusarium oxysporum* SZMC 6237 J (Vegetables/Hungary)	**25**	> 400	**25**
*Fusarium proliferatum* SZMC 21643 (Corn/Hungary)	**25**	n.d.	n.d.
*Fusarium sporotrichoioides* SZMC 11043 (n.d.)	**25**	n.d.	n.d.
*Fusarium verticillioides* SZMC 21642 (Corn/Hungary)	**25**	n.d.	n.d.
*Trichoderma harzianum* SZMC 1601 (Wheat rhizosphere/Hungary)	**25**	n.d.	n.d.

Bold numerical values represent the MICs

*CBS* Centraalbureau voor Schimmelcultures, Utrecht, The Netherlands, *FSU* Fungal Reference Center University of Jena, Jena, Germany, *SZMC* Szeged Microbiological Collection, University of Szeged, Szeged, Hungary, *n.d.* data not available

### Cultivation of Tomato Plants

Tomato (*S. lycopersicum* L. cv. Ailsa Craig) seeds were grown in darkness at 27 °C for 3 days, then transferred to Perlite (bulk density: 90–110 kg m^–3^, particle size: 3–6 mm, moisture content: >2%, pH = 6.0–7.5; Agrolit Kft., Olaszliszka, Hungary) and maintained for 2 weeks. The plants were grown hydroponically for 4 weeks under controlled environmental conditions, maintaining 200 µmol m^−2^ s^−1^ photon flux density, 12/12 h light/dark photoperiod, day/night temperatures of 23 °C/20 °C, and 50%–60% relative humidity, following the protocol established by Poór et al. [37].

### In Silico Investigations

The physicochemical properties of native and recombinant K4CBP6 and its γ-core peptide derivatives were predicted using ExPASy ProtParam [38], while the total net charge at pH 7.0 was estimated via Protein Calculator v3.4 (The Scripps Research Institute; https://protcalc.sourceforge.net/). The cleavage site of the signal sequence was predicted with SignalP 4.0 [39]. A homology model of K4CBP6 was generated using I-TASSER [40] and refined via energy minimization using ModRefiner [41]. The reliability of the predicted structure was assessed via Ramachandran plot analysis using the RAMPAGE server [42]. The disulfide bridge pattern was estimated using the DISULFIND server [43]. The tertiary structure, hydrophobicity, and charge surface were visualized using the UCSF Chimera software [44]. The Basic Local Alignment Search Tool (BLAST) function of UniProt was used to screen for K4CBP6 homologs [45]. Additionally, BioEdit [46] was employed to examine the sequence of defensins.

### Heterologous Expression of rK4CBP6 in *K. Phaffii*

The methanol-induced extracellular production of rK4CBP6 was conducted in *K. phaffii* KM71H using the pPICZαA expression vector (EasySelect Pichia Expression Kit, Invitrogen-Life Technologies, Carlsbad, CA, USA). The codon-optimized cDNA of K4CBP6, designed for expression in *K. phaffii*, along with the corresponding primers (K4CBP6F and K4CBP6R), was synthesized by Eurofins Genomics, Inc. (Ebersberg, Germany). The 182 bp cDNA fragment, embedded in the pEX-A128-K4CBP6new_Pichia plasmid, was amplified using the primer pair K4CBP6F (5′-GGC CCT CGA GAA AAG AGA GGC TGA AGC T-3′) and K4CBP6R (5′-GGC CTC TAG ATT AGC ATG GCT T-3′). The forward primer (K4CBP6F) incorporates the *Xho*I restriction site (CTC GAG), the Kex2 protease cleavage site (GAG AAA AGA), and a Glu-Ala repeat (GAG GCT GAA GCT) containing the Ste13 cleavage site, all positioned at the N-terminal region of the construct. The reverse primer (K4CBP6R) includes the *Xba*I restriction site (TCT AGA) at the C-terminal end (Fig. S1). PCR amplification was performed under the following conditions: initial denaturation at 94 °C for 1 min 30 s; followed by 30 cycles of 94 °C for 10 s, 60 °C for 15 s, and 72 °C for 20 s; with final extension at 72 °C for 5 min. The amplified cDNA and pPICZαA expression vector were digested with *Xba*I and *Xho*I restriction endonucleases (Thermo Scientific, Waltham, MA, USA) and ligated using T4 DNA ligase (Thermo Scientific, Waltham, MA, USA). The resulting vector construct pPICZαA_K4CBP6 was transformed into *Escherichia coli* TOP 10 F⁻ cells [47]. pPICZαA_K4CBP6 was purified using the ZymoPURE™ Express Plasmid Midiprep Kit (Zymo Research, Irvine, CA, USA) from *E. coli* TOP 10 F⁻ transformants. The purified plasmid was digested with *Sac*I (Thermo Scientific, Waltham, MA, USA). The digested vector construct was introduced into *K. phaffii* KM71H cells using the lithium chloride transformation method, according to the manufacturer’s instructions (EasySelect Pichia Expression Kit, Invitrogen-Life Technologies, Carlsbad, CA, USA).

### rK4CBP6 Production and Purification

rK4CBP6 production was induced in *K. phaffii* KM71H cultures by adding 0.5% methanol (v/v) for 4 days at 30 °C with continuous shaking at 160 rpm. Following fermentation, the cells were separated from the medium via centrifugation (5 min, 3,000 × *g*, 25 °C). The cell-free supernatant was diluted sixfold with distilled water prior to cation-exchange chromatography using a Bio-Scale™ Mini Macro-Prep High S column (Bio-Rad Laboratories, Hercules, CA, USA) with an NGC Medium-Pressure Liquid Chromatography instrument (Bio-Rad Laboratories, Hercules, CA, USA). The chromatography column was equilibrated with 10 mM Na₂HPO₄/NaH₂PO₄ buffer (pH 6.6) containing 25 mM NaCl and 0.15 mM EDTA. Bound proteins were eluted with increasing NaCl concentrations (0.1 M, 0.2 M, 0.3 M, and 1.5 M) prepared in 10 mM Na₂HPO₄/NaH₂PO₄ buffer (pH 6.6) at a flow rate of 2 ml min^–1^. The rK4CBP6-containing fractions were analyzed using SDS-PAGE (18% Tris-Glycine gel, 1.0 mm, 10-well). Protein bands were visualized with Coomassie Brilliant Blue R-250 staining. The pure rK4CBP6 fractions were dialyzed in a SnakeSkin™ dialysis tubing (3.5 K MWCO, Thermo Fisher Scientific, Waltham, MA, USA) against distilled water at a ratio of 1:200 for 8 h at room temperature. All production and purification experiments were repeated six times to ensure consistency and reliability of the rK4CBP6 yield.

### Electrospray Ionization Mass Spectrometry (ESI-MS) Analysis

The molar mass of rK4CBP6 was determined using an Orbitrap mass spectrometer (Thermo Q-Exactive+, Thermo Fisher Scientific, Waltham, MA, USA) connected to an ultra-high-performance liquid chromatography system (Nano Equity Ultraperformance Liquid Chromatography System, Waters, Milford, MA, USA). For protein mass measurement, a sample collected as an analytical high-performance liquid chromatography fraction was injected into the eluent using the flow injection method. The eluent consisted of a mixture of (0.1% (v/v) formic acid (FA) in 50:50% (v/v) water and acetonitrile (ACN).

### Reversed-Phase High-Performance Liquid Chromatography (RP-HPLC) Purification and Analysis

Although cation exchange chromatography yielded a high-purity preparation of rK4CBP6, residual contaminants (including salts, sugars, lipids, and other minor impurities) may still be present. Therefore, to enable detailed structural and functional characterization and to achieve near-homogeneous purity, further RP-HPLC purification step, was performed using the following solvent system: eluent A, 0.1% (v/v) trifluoroacetic acid (TFA); and eluent B, 0.1% (v/v) TFA in 80% (v/v) ACN. Purification was performed on a Phenomenex Jupiter C18 column (250 × 10 mm, 10 μm particle size, 300 Å pore size; Phenomenex, Torrance, CA, USA) using an Agilent-Shimadzu apparatus (Agilent Technologies, Santa Clara, CA, USA). The eluent B concentration was gradually increased from 0% to 30% (v/v) at a flow rate of 3 ml min^–1^ over 60 min, ensuring precise separation and purification of rK4CBP6. The purified protein was analyzed on a Phenomenex Jupiter C18 column (250 × 4.6 mm, 10 μm particle size, 300 Å pore size; Phenomenex, Torrance, CA, USA) using an Agilent 1100 Series liquid chromatograph (Agilent Technologies, Santa Clara, CA, USA). A linear gradient from 20% to 35% (v/v) eluent B was applied at a flow rate of 1 ml min^–1^ over 15 min.

### Structural Investigation with Electronic Circular Dichroism (ECD)

The secondary structure and thermal stability of rK4CBP6 were evaluated using a Jasco-J815 ECD spectrometer (JASCO, Tokyo, Japan) in the far-UV range (185–260 nm). rK4CBP6 was dissolved in pure water at a final concentration of 0.1 mg ml^− 1^ and measured in a 0.1 cm pathlength quartz cuvette. The temperature was set using a Peltier thermoelectric controller (TE Technology, Traverse City, MI, USA). The spectrum of pure water was subtracted from the spectra of rK4CBP6. The presented spectra are accumulations of 10 scans and reported in mean residue molar ellipticity units ensuring the accurate assessment of protein conformational properties.

### Synthesis, Purification, and Analysis of K4CBP6 γ-core Peptide Derivatives

K4CBP6γ1 and K4CBP6γ2 peptide derivatives, spanning the γ-core motifs of K4CBP6, were rationally designed and chemically synthesized using Fmoc-based solid-phase peptide synthesis, following the methodology described by Sonderegger et al. [48] (Table 2). Purification and analysis were performed utilizing the same solvent system and the same RP-HPLC equipment as the protein (paragraph 3.7.). The peptides were purified on a Phenomenex Jupiter C18 column (250 × 10 mm, 10 μm particle size, 90 Å pore size; Phenomenex, Torrance, CA, USA) applying a linear gradient from 0% to 30% (v/v) eluent B at a flow rate of 3 ml min^–1^ over 60 min. The purified peptides were analyzed on a Phenomenex Luna C18 column (250 × 4.6 mm, 10 μm particle size, 100 Å pore size; Phenomenex, Torrance, CA, USA) at a flow rate of 1 ml min^–1^. The 15-min linear gradients were as follows: from 18% to 33% (v/v) eluent B for K4CBP6γ1 and from 21% to 36% (v/v) eluent B for K4CBP6γ2.

**Table 2:** Amino acid sequence and in silico predicted physicochemical properties of K4CBP6 from *Solanum lycopersicum* L., its γ-core motifs (γ1 and γ2), the recombinant K4CBP6 (rK4CBP6) produced in *Komagataella phaffii*, and the synthesized γ-core peptide derivatives (K4CBP6γ1 and K4CBP6γ2)

Protein	Number of amino acids	Mw. (kDa)	Number of cysteines	Lysine/Arginine/Histidine	Isoelectric point	Charge (pH 7)	GRAVY
*MAQSIRFFATLFLLAMLVMATEMGPTRIVEA* **RHCESLSHRFK** **GPCVSDKNC** **ASVCETERFSG** **GNCRGFRRRC** **FCTKPC**							
**K4CBP6**	47	5.4	8	3/7/2	9.14	+ 6.2	−0.757
GPCVSDKNC							
**γ1**	9	0.9	2	1/0/0	5.82	−0.2	−0.500
GNCRGFRRRC							
**γ2**	10	1.2	2	0/4/0	11.53	+ 3.8	−1.450

**rK4CBP6**	51	5.7	8	3/7/2	8.76	+ 4.2	−0.765
Ac-RFKGPC(-SH)VSDKNC(-SH)K-NH2							
**K4CBP6γ1**	13	1.48	2	3/1/0	9.39	+ 2.8	−1.077
Ac-FSGGNC(-SH)RGFRRRC(-SH)K-NH2							
**K4CBP6γ2**	14	1.64	2	1/4/0	11.54	+ 4.8	−1.200

In the amino acid sequence of K4CBP6, italic residues represent the prepro-sequence, whereas bold residues correspond to the mature protein. The γ-core motifs within the primary structure are denoted by underlined bold residues. Additionally, the amino acid sequence of rK4CBP6 contains a Glu-Ala (EAEA) repeat motif, highlighted by a black frame. Mw: molecular weight, GRAVY: grand average of hydropathy value

### In Vitro Antifungal Susceptibility Tests

The broth microdilution susceptibility testing method described by Tóth et al. [49] was employed to determine the minimum inhibitory concentrations (MICs) of rK4CBP6 and its γ-core peptide derivatives against plant pathogenic fungi. Conidial suspensions were prepared by harvesting conidia from 7-day-old cultures grown on PDA in T-25 polystyrene cell culture flasks (Sarstedt, Nümbrecht, Germany), with the exception of *B. cinerea* SZMC 21472, which was collected from 10-day-old cultures. Conidia were collected using an inoculation loop in spore buffer (SB; 0.9% NaCl (m/v), 0.01% Tween 80 (v/v)) and filtered through a sterile 40 μm cell strainer (VWR, Radnor, PA, USA). The filtrate was centrifuged at 4,000 × g for 5 min, and the resulting conidial pellet was washed three times with SB. Washed conidia were resuspended in SB, counted using a Bürker chamber, and diluted to 2 × 10⁵ conidia ml^–1^ in 0.1× PDB. Susceptibility tests were conducted in 96-well, flat-bottom microtiter plates (Tissue Culture Plates, VWR, Radnor, PA, USA). For this, 100 µl of rK4CBP6, K4CBP6γ1, or K4CBP6γ2 solution (concentration range: 0.39–400 µg ml^–1^, twofold dilutions in 0.1 × PDB) was mixed with 100 µl of 2 × 10⁵ conidia ml^− 1^ suspension, prepared in 0.1 × PDB. Growth control wells contained 100 µl of 0.1 × PDB mixed with 100 µl of 2 × 10⁵ conidia ml^− 1^. The plates were incubated at 24 °C for 72 h without shaking. Fungal growth was evaluated by measuring the optical density at 620 nm (OD₆₂₀) using a Microplate Reader 96 & 384 Thermo Multiskan Ascent (Thermo Fisher Scientific, Waltham, MA, USA). Fresh medium (200 µl of 0.1 × PDB) was used for background calibration. The untreated control culture was set to 100% growth, and treated samples were compared to the control to determine fungal inhibition. The MIC was defined as the lowest concentration of antifungal protein or peptide at which fungal growth was ≤ 5% after 72 h of incubation, based on OD₆₂₀ values compared to the untreated control. All microdilution experiments were performed at least twice, with two technical replicates to ensure reproducibility and accuracy.

### *Galleria Mellonella* Toxicity Assay

The in vivo toxicity effects of rK4CBP6 and K4CBP6γ2 were investigated using *G. mellonella* larvae (ChameleonFarm, Budapest, Hungary). Twenty larvae were used in each treatment and control group. rK4CBP6 and K4CBP6γ2 solutions (200 µg ml^− 1^) were prepared in insect physiological saline (IPS: 50 mM NaCl, 5 mM KCl, 10 mM EDTA, and 30 mM sodium citrate in 0.1 M Tris-HCl; pH 6.9), and 20 µl of these solutions were injected (29-gauge insulin needles; BD Micro-Fine, Franklin Lakes, NJ, USA) intrahemocoelically through one of the last two pro-legs of the larvae. Larvae were incubated at 37 °C, and their survival was monitored daily for 6 days. A larva was considered dead if it showed no movement in response to touch. IPS-treated and untreated larvae served as negative controls, while larvae injected with 20% (v/v) Triton X-100 comprised the positive toxicity control group. All toxicity tests were repeated three times.

### Hemolysis Assays

The hemolytic activity of rK4CBP6 and K4CBP6γ2 was evaluated using Columbia sheep blood agar plates (VWR, Radnor, PA, USA). Sterile paper discs (Ø 6 mm) were placed on the agar surface, and 10 µl of rK4CBP6 or K4CBP6γ2 (1 mg ml^− 1^) was subsequently pipetted onto the discs and allowed to dry at room temperature. 20% (v/v) Triton X-100 was used as the positive control, while sterile distilled water served as the negative control. The plates were incubated at 37 °C for 24 h. Hemolytic activity was assessed by monitoring the presence of clear zones around the discs, indicating erythrocyte lysis.

Hemolytic activity of the compounds was also tested on human red blood cells (RBCs), obtained from healthy volunteers collected by the Hungarian National Blood Transfusion Service (Budapest, Hungary). First, RBCs were washed twice with phosphate buffered saline (PBS), then diluted to 2% (v/v) in PBS, and plated on a 96-well, round-bottom plate (Tissue Culture Plates, VWR, Radnor, PA, USA) at a volume of 75 µl per well. Serial dilutions of the compounds were prepared at a volume of 75 µl and added to the erythrocytes (final concentrations: 0.8–200 µg ml^− 1^). The final RBC concentration was adjusted to 1% (v/v), with a final volume of 150 µl in the wells. Plates were incubated at 37 °C in 5% CO_2_ for 2 h, then centrifuged (2,000 rpm, 5 min, 4 °C). Subsequently, 50 µl of the supernatants were carefully transferred to a new plate containing 100 µl H_2_O in each well. Optical density was measured at 414 and 450 nm using a Thermo Multiscan GO plate reader (Thermo Fisher Scientific, Waltham, MA, USA). Percentage of hemolysis was compared to the positive control, the bee venom Melittin (10 µM, in PBS). All data are presented as the mean ± standard deviation (SD) (*n* = 3).

### Toxicity Tests on Mammalian Cells

Three human model cell lines were chosen to characterize the cytotoxicity of the compounds, THP1 (ATCC: TIB-201), HT29 (ATCC No.: HTB-38), and HepG2 (ATCC No.: HB-8065). Prior to treatment, cells were cultured for 24 h in 10% FBS containing RPMI-1640 medium (20,000 THP1 cells, or 5,000 HT29 and HepG2 cells/100 µl/well, in a flat-bottom 96-well culture plate). Stock solutions of the compounds were diluted with media, and a three-fold serial dilution series was prepared (final concentrations: 0.8–200 µg ml^− 1^). Cells were treated with the compounds for 24 h, washed twice with media, and then, in the case of HT29 and HepG2, incubated for further 3 days. In the case of the THP1, cell viability was tested immediately after washing, using the colorimetric MTT test [50]. Briefly, 45 µl MTT solution (2 mg ml^− 1^, dissolved in serum-free medium) was added to each well. After 3 h of incubation, the plates were centrifuged at 2,000 rpm for 5 min, and the supernatant was carefully aspirated. The precipitated purple crystals were dissolved in 100 µl DMSO, and after 5 min of agitation, the absorbance was determined at 550 nm and 660 nm using a Thermo Multiscan GO plate reader. Cytotoxicity, compared to RPMI media-treated control cells, was expressed in percentage as a function of the compound concentrations, and presented as the mean ± SD (*n* = 3).

### Toxicity Tests on Plant Seedlings

The potential toxic effects of rK4CBP6 and K4CBP6γ2 were investigated in seedlings of *Medicago truncatula* A-17 and *S. lycopersicum* L. cv. Ailsa Craig using controlled experimental conditions. *M. truncatula* seeds were treated with 96% (v/v) sulfuric acid for 5 min, followed by three washes with cold distilled water. The sterilized seeds were plated on 1% (w/v) water agar (Agar HP 696; Kalys, Bernin, France) and germinated in darkness at 4 °C for 3 days, and at 25 °C for additional 24 h. *S. lycopersicum* seeds were sterilized using 70% (v/v) ethanol for 20 s, followed by 5% (v/v) sodium hypochlorite treatment for 10 min. Finally, seeds were washed with distilled water three times for 20 min. Subsequently, they were plated on 1% (w/v) water agar and germinated in darkness at 4 °C for 3 days, and at 25 °C for additional 3 days. Five germinated seeds were placed in a single lane on water agar medium prepared in square Petri dishes for each treatment. In the case of *M. truncatula*, seeds were placed 20 mm from the top in 120 × 120 × 17 mm Bio-One Square Petri Dishes (Greiner, Sigma-Aldrich, St. Louis, MO, USA), while *S. lycopersicum* seeds were placed 80 mm from the top in 245 × 245 × 25 mm Nunc Square BioAssay dishes (Thermo Scientific, Waltham, MA, USA). The apical region of primary roots was treated daily with 20 µl of rK4CBP6 or K4CBP6γ2 dissolved in water, at concentrations of 200 µg ml^− 1^ for *M. truncatula* and 400 µg ml^− 1^ for *S. lycopersicum*. Following treatment, the root regions were covered with aluminum foil to maintain darkness. Plates were incubated in a humid (60%) plant growth chamber under photoperiodic day-night simulation (12/12 h with or without illumination at 1200 lx) at 23 °C. After 6 days for *S. lycopersicum* and 10 days for *M. truncatula*, the length of primary roots and number of lateral roots were calculated. Distilled water-treated seedlings were used as growth controls. Ethanol-treated seedlings (70% (v/v)) served as dead controls. Toxicity tests were repeated four times.

### Plant Toxicity Assay

The potential harmful effects of rK4CBP6 and K4CBP6γ2 were assessed in intact tomato plants (*S. lycopersicum* L. cv. Moneymaker) following the methodology of Tóth et al. [51, 52]. Ten microliters of rK4CBP6 and K4CBP6γ2 at 400 µg ml^− 1^ and 25 µg ml^− 1^, respectively, was applied at five points between the left lateral veins on the abaxial leaf epidermis of five fully expanded leaves at the third leaf level. A 10% (v/v) Tween80 was used as a positive control, while untreated leaves served as a negative control. Plants were maintained in a humid (60%) plant growth chamber at 23 °C under photoperiodic day-night simulation (12/12 h with or without illumination at 1200 lx) for 4 days. After incubation, Evan’s blue staining was performed to visualize necrotic zones around the treatment points on detached leaves, according to the method described by Tóth et al. [51]. Images of stained leaves were obtained using a Canon EOS 700D camera (Tokyo, Japan). For each treatment, five leaves were used, and the plant toxicity assays were repeated at least twice.

### Plant Protection Experiment

The plant protection assay was conducted following the methodology described by Tóth et al. [51] to evaluate the protective effect of rK4CBP6 (25 µg ml^− 1^) and K4CBP6γ2 (25 µg ml^− 1^) against *Botrytis cinerea* SZMC 21472 on the leaves of *S. lycopersicum* L. cv. Moneymaker. For the investigation of protective effect of rK4CBP6 and K4CBP6γ2, 10 µl of *B. cinerea* SZMC 21472 conidial suspension (1 × 10⁷ conidia ml^− 1^) containing 25 µg ml^− 1^ defensin or peptide, prepared in 0.1 × PDB, was applied to the abaxial leaf epidermis of detached leaves from six-week-old plants at three points between the lateral veins. To assess infection suppression, necrotic zones around treatment points were visualized using Evan’s blue staining, following the protocol of Tóth et al. [51]. Images of stained leaves were obtained using a Canon EOS 700D camera (Tokyo, Japan). Plant protection experiments were repeated at least three times, with three leaves per treatment.

### Fruit Protection Experiment

The toxicity and fruit protection potential of rK4CBP6 and K4CBP6γ2 were evaluated using tomato fruits (“On The Vine Red” variety) from a local supermarket, following the methodology described by Tóth et al. [52, 53]. Tomato fruits were pierced to a depth of 3 mm at three points near the stalk. For infection control, 10 µl of *Cladosporium herbarum* FSU 1148 conidial suspension (1 × 10^7^ conidia ml^–1^), and for toxicity testing, 10 µl of rK4CBP6 (25 µg ml^–1^) or K4CBP6γ2 (12.5 µg ml^–1^) was used. For the fruit protection assay, 10 µl of *C. herbarum* FSU 1148 conidial suspension (1 × 10^7^ conidia ml^–1^) containing 25 µg ml^–1^ of rK4CBP6 or 12.5 µg ml^–1^ of K4CBP6γ2 was used. As an uninfected control, 10 µl of 0.1 × PDB was pipetted into the holes. All treatments were allowed to dry at room temperature. Tomato fruits without punctures were used as untreated controls. After treatment, tomato fruits were incubated in a sterile plastic box at room temperature for 1 week. A humid atmosphere was provided and maintained by attaching a wet paper towel to the inner side of the plastic box lid. Following incubation, the fruits were axially cut in half to assess tissue damage and the presence and extent of fungal infection. Images of tomato fruits were obtained using a Canon EOS 700D camera (Tokyo, Japan). All experiments were repeated at least twice, and three fruits were used for each treatment.

### Statistical Analyses

Means and standard deviations were calculated using Microsoft Excel 2016 (Microsoft, Edmond, WA, USA). One-way ANOVA and Tukey’s HSD post-hoc tests were performed using the Statistics Kingdom online platform (Statistics Kingdom, 2021) to analyze differences among experimental groups. Differences were considered statistically significant at *p* ≤ 0.05. *G. mellonella* survival curves were statistically compared using the log-rank (Mantel–Cox) and Gehan–Breslow–Wilcoxon tests in GraphPad Prism 7.00 (GraphPad Software, Boston, MA, USA). The toxic effect of the defensin or peptide was considered significant if *p* ≤ 0.05 in both tests.

Table S1 provides a summary of the equipment used in the experiments, including their names, manufacturers, and technical specifications.

## Results

### In Silico Characterization of K4CBP6

Based on in silico predictions, K4CBP6 is expressed as a 78-amino acid preprotein, containing a 31-amino acid N-terminal signal sequence that is cleaved during maturation process (Table 2). Consequently, the mature K4CBP6 comprises 47 amino acids, with a calculated molecular weight of 5357.14 Da and an isoelectric point of 9.14 (Table 2). K4CBP6 is positively charged (net charge at pH 7.0 = + 6.2) and hydrophilic (GRAVY = − 0.757). Its tertiary structure is stabilized by four intramolecular disulfide bridges between eight cysteine residues in an *abcdbcda* bonding pattern (C3–C47, C14–C34, C20–C41, and C24–C43). This disulfide bonding pattern is a common structural feature among plant defensins [54] (Table 2).

**Fig. 1:**
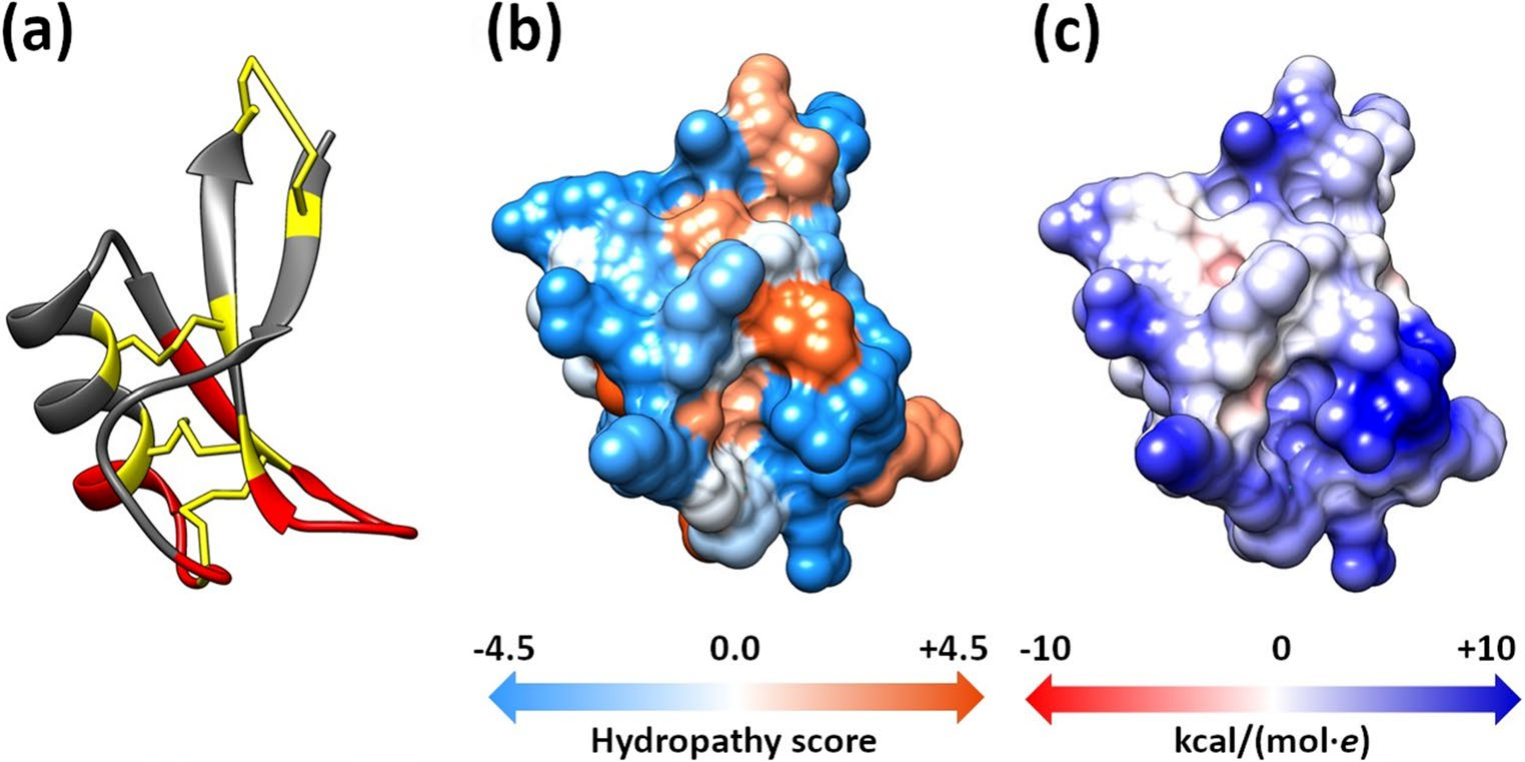
The predicted (**a**) tertiary structure, (**b**) surface hydrophobicity, and (**c**) surface charge distribution of K4CBP6 generated using in silico homology modeling. Cysteine residues are marked in yellow, while the disulfide bridges formed between them are represented by yellow lines. The γ-core regions within the peptide structure are highlighted in red. The energetically optimal conformation of each amino acid side chain in the modeled structure was verified using Ramachandran plot analysis, confirming that 100% of the amino acids in K4CBP6 occupy the most energetically favored region. This finding suggests that the predicted structural model is highly reliable. The tertiary structure of K4CBP6 consists of one α-helix and three antiparallel β-sheets (**a**). The surface is amphipathic, with alternating hydrophilic and hydrophobic regions (**b**), and is dominated by neutral and positively charged areas (**c**)

The predicted tertiary structure of K4CBP6 closely resembles that of other plant defensins [22, 23]. It comprises an α-helix and three antiparallel β-sheets (Fig. 1a). The surface of K4CBP6 is amphipathic (Fig. 1b), characterized by alternating hydrophilic and hydrophobic regions, and it predominantly contains neutral and highly positively charged regions (Fig. 1c). The predicted structural model, validated by Ramachandran plot analysis, demonstrates high reliability and structural integrity, with 100% of the residues in energetically favored region (data not shown).

### Homologs of K4CBP6

To determine whether K4CBP6 is unique to *S. lycopersicum* L. or is evolutionarily conserved and widespread across the plant kingdom, a homology search was conducted using the UniProt database [42]. Thirty plant defensins were identified, exhibiting high similarity to K4CBP6 (Fig. S2) with 92%–71% identity at the amino acid level (Table S2). The most similar sequences (>81% identity) were found within the genus *Solanum*. However, interestingly, the highest number of homologs was observed in *Nicotiana tabacum*, with 73%–77% identity (Table S2). Moreover, three homologs were identified in *Solanum tuberosum*, *Nicotiana sylvestris*, *Solanum commersonii*, and *Solanum verrucosum*. In *Anisodus tanguticus*, *Capsicum annuum*, and *Capsicum baccatum*, two similar defensins were detected. Furthermore, in six additional species (*Anisodus acutangulus*, *S. lycopersicum*, *Vigna angularis* var. *angularis*, *Phaseolus vulgaris*, *Vigna radiata* var. *radiata*, and *Cajanus cajan*), only one homolog was identified (Table S2). Interestingly, the UniProt search identified the K4CBP6 as a knottin scorpion toxin-like domain-containing protein in *S. lycopersicum* (Table S2). To date, none of the knottin homologs identified in *Solanum* species or other plants have been experimentally characterized, making this study the first to provide a functional description of this distinct defensin lineage.

These findings suggest that K4CBP6 exhibits a degree of evolutionary conservation, particularly among species of the Solanaceae family, and may play a broader role in plant defense mechanisms.

### rK4CBP6 Production in *K. Phaffii*

Antifungal plant defensins are increasingly recognized as effective biofungicide molecules against phytopathogenic filamentous fungi. However, their large-scale application as biofungicides is hindered by the lack of an optimized, cost-effective industrial production system [14, 55]. The low natural abundance of defensins within host organisms and the complexity of isolation process pose significant obstacles to determining their antifungal spectrum, mechanism of action, and potential toxicity across different organisms. To overcome these limitations, establishing a reliable heterologous expression system is crucial [14].

**Fig. 2:**
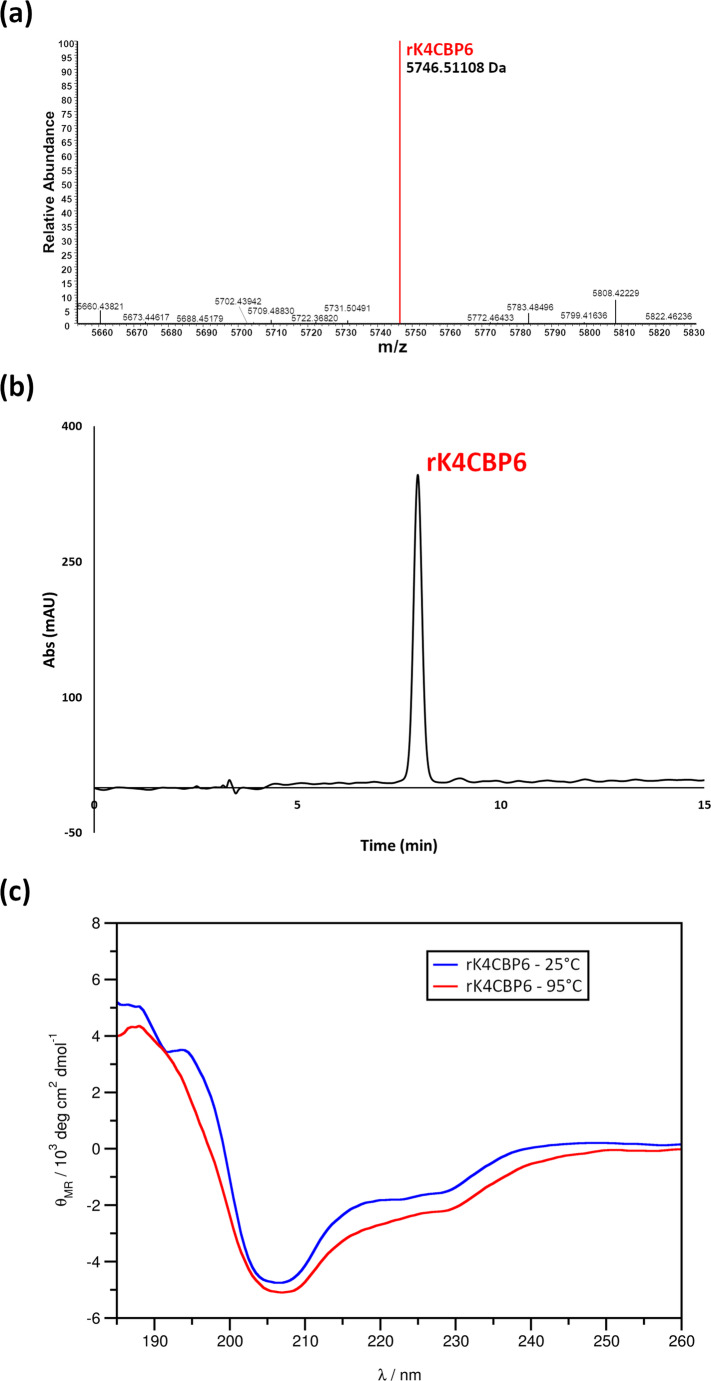
(**a**) Electrospray ionization mass spectrometry spectrum, (**b**) reversed-phase high-performance liquid chromatography chromatogram (RP-HPLC), and (**c**) electronic circular dichroism spectra of the purified rK4CBP6. A linear gradient of solvent B (22%–37% (v/v)) was applied over 15 min for the RP-HPLC analysis. rK4CBP6: recombinant K4CBP6 produced by *Komagataella phaffii*. rK4CBP6 25 °C and 95 °C: spectra of recombinant K4CBP6 recorded at 25 °C and 95 °C. rK4CBP6 is a 5746 Da protein containing four intramolecular disulfide bonds (**a**) and adopting in a single, stable tertiary conformation (**b**). ECD analysis confirmed that the structure of rK4CBP6 is heat-stable and contains secondary elements typical of plant defensins (α-helix, β-sheets, and random structures) (**c**)

A methanol-inducible *K. phaffii*-based expression system is widely employed for large-scale production of various plant defensins [55–59]. rK4CBP6 was successfully expressed in *K. phaffii* KM71H using this system, with a yield of 7.94 ± 1.25 mg l^− 1^ (*n* = 6). ESI-MS revealed a monoisotopic molecular weight of 5746 Da (Fig. 2a), which exceeded the calculated weight of 5357 Da. This discrepancy is attributed to the presence of an EAEA repeat from the signal sequence, likely resulting from improper cleavage. Moreover, ESI-MS confirmed the presence of four intramolecular disulfide bonds (− 2 Da per bond) between eight cysteine residues (Fig. 2a). The tertiary structure of rK4CBP6 was examined via RP-HPLC, which displayed a single peak in the chromatogram, indicating that rK4CBP6 exists in a single stable tertiary conformation (Fig. 2b).

### Structural Investigation of rK4CBP6

ECD spectroscopy was used to characterize the secondary structural elements of rK4CBP6. The ECD spectrum at 25 °C revealed a mixed α-helical and β-sheet secondary structure. The maximum at 195 nm indicates β-sheet structures, whereas the maximum at 190 nm, along with the broad double minimum at 206 nm and 225 nm, reflects α-helical content. The broadening of the double minimum and the slight shift of minima from typical α-helical values (208 and 222 nm, respectively) indicate the presence of unordered structural elements. The spectral properties at both 25 °C and 95 °C are highly similar, demonstrating the remarkable thermal stability of the rK4CBP6 defensin (Fig. 2c). ECD analysis confirmed that rK4CBP6 adopts a folded conformation and that the EAEA repeat does not impede protein maturation. However, elucidation of its precise three-dimensional structure would require further characterization by nuclear magnetic resonance spectroscopy. The presence of typical secondary structural elements (α-helix, β-sheets, and random structures) aligns with the structural characteristics of plant defensins [21, 22].

### Synthesis of K4CBP6 γ-core Peptide Derivatives

Several studies have reported the antifungal activity of peptides synthesized based on the γ-core regions of plant defensins [27]. Rigano et al. [51] has demonstrated the antibacterial activity of the chemically synthesized γ-core motif of K4CBP6 (SolyC). However, the antifungal properties of this peptide have been less extensively explored, although the γ-core motifs with the RGFRRR signature have been studied in several reports [27, 28, 60]. To investigate the antifungal activity of the K4CBP6 γ-core motif, two peptides spanning the two γ-core regions, K4CBP6γ1 and K4CBP6γ2, were rationally designed and chemically synthesized. The native γ-core motifs of K4CBP6 are located within the β2-loop-β3-sheet region of plant defensins [22, 60]. The γ1 region primarily comprises neutral and slightly hydrophilic residues (net charge at pH 7.0 = − 0.2, GRAVY = − 0.500), while the γ2 region is positively charged and highly hydrophilic (net charge at pH 7.0 = + 3.8, GRAVY = − 1.450) (Table 2). Both peptides were designed according to the method of Sonderegger et al. [45]. They possess three additional N-terminal amino acids and an extra lysine residue at the C-terminus. Furthermore, N-terminal acetylation and C-terminal amidation were applied to mimic the native protein structure, neutralize terminal charges, and enhance proteolytic stability. The chemically synthesized K4CBP6 γ-core peptide derivatives, K4CBP6γ1 and K4CBP6γ2, exhibit positive net charges (+ 2.8 and + 4.8 at pH 7.0, respectively) and hydrophilic properties (GRAVY = − 1.077 and − 1.200, respectively) (Table 2).

### Antifungal Activities of rK4CBP6 and γ-core Peptide Derivatives

The antifungal activities of rK4CBP6 and its synthetic γ-core peptide derivatives, K4CBP6γ1 and K4CBP6γ2, were assessed using broth microdilution susceptibility tests against various plant pathogenic fungi. The minimum inhibitory concentrations (MICs) observed are summarized in Table 1. rK4CBP6 effectively inhibited the growth of all tested *Fusarium* isolates, except for *Fusarium subglutinans*, with MICs ranging from 25 to 50 µg ml^− 1^. Moreover, rK4CBP6 demonstrated antifungal activity against *B. cinerea* and *C. herbarum*, with a MIC of 25 µg ml^− 1^. However, rK4CBP6 was ineffective against *Aspergillus* isolates within the tested concentration range. The antifungal properties of K4CBP6γ1 and K4CBP6γ2 were evaluated against three key pathogens of tomato plants, *B. cinerea*, *C. herbarum*, and *F. oxysporum*. K4CBP6γ1 showed no inhibitory effect on *B. cinerea* and *F. oxysporum* within the tested concentration range. However, it was able to inhibit the growth of *C. herbarum*, with a MIC of 400 µg ml^–l^. K4CBP6γ2 exhibited strong antifungal activity, effectively inhibiting the growth of all tested fungi, with MICs ranging from 12.5 µg ml^− 1^ to 25 µg ml^− 1^. These findings highlight the variable antifungal efficacy of rK4CBP6 and its γ-core peptide derivatives, emphasizing the potential of K4CBP6γ2 as a promising antifungal agent for plant protection.

Based on these findings, further experiments were conducted using only the rK4CBP6 defensin and the K4CBP6γ2 peptide owing to their superior antifungal potency.

### In Vitro and in Vivo Toxicity of rK4CBP6 and K4CBP6γ2

The above findings indicate the potential of rK4CBP6 and K4CBP6γ2 as biofungicides for controlling phytopathogenic fungi. However, comprehensive toxicity assessments on non-target organisms (such as animals) are necessary to ensure their safe application.

**Fig. 3:**
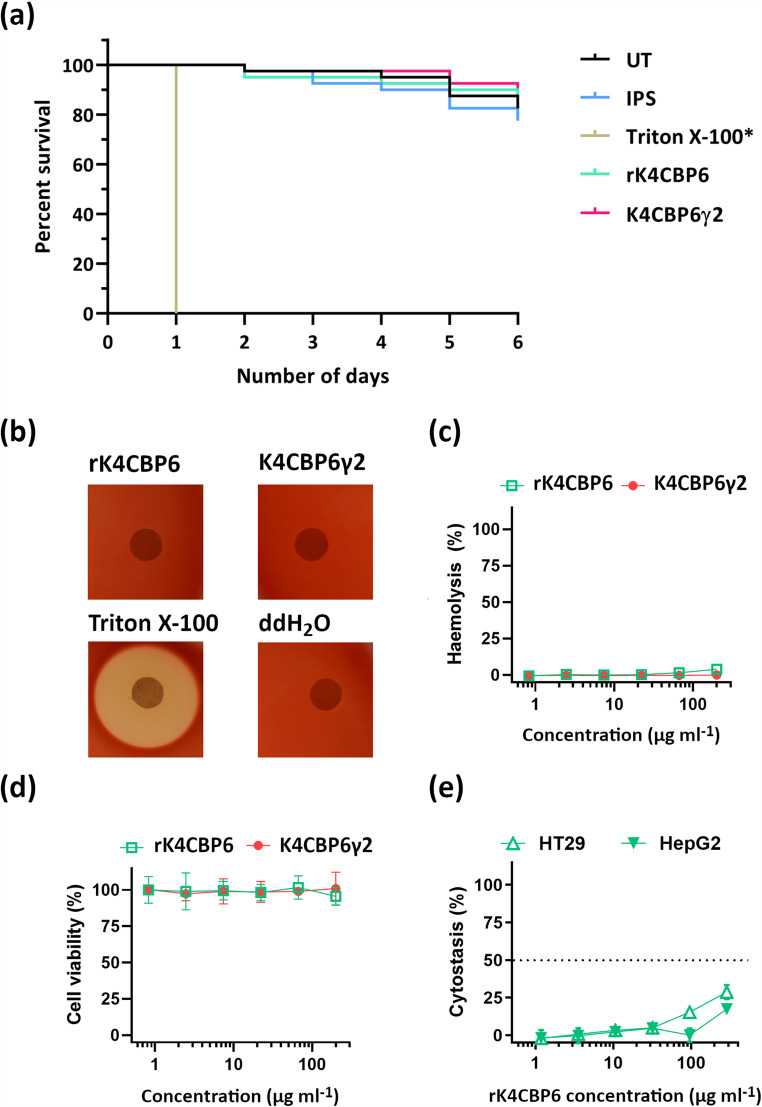
(**a**) Survival analysis of *Galleria mellonella* larvae following treatment with rK4CBP6 and K4CBP6γ2 (20 µl of 200 µg ml^− 1^ solutions) compared to the untreated control. UT: untreated control, IPS: insect physiological saline-treated control. Statistically significant differences were determined using log-rank (Mantel–Cox) and Gehan–Breslow–Wilcoxon tests, with significance indicated by * (*p* ≤ 0.05; *n* = 60). (**b**) Hemolytic activity of rK4CBP6 and K4CBP6γ2 in aqueous solution on Columbia sheep blood agar plates following 24-h incubation at 37 °C. Triton X-100 (20% (v/v)) was used as the positive control, while distilled water (H_2_O) served as the negative control. (**c**) Hemolytic activity of rK4CBP6 and K4CBP6γ2, dissolved in PBS, on human red blood cells following 2-h incubation. (**d**) Viability of THP1 human monocytes after 24-h incubation with rK4CBP6 and K4CBP6γ2 compounds in RPMI-1640 media. (**e**) Long-term cytostatic effect of rK4CBP6 on HT29 human colorectal and HepG2 human hepatocellular cell line. Twenty-four-hour treatment with the compound was followed by 3 days of incubation in 10% FBS containing RPMI-1640 media, and then viability of the cells was evaluated by colorimetric MTT test. All data are represented as the mean ± SD (*n* = 3). Treatment with rK4CBP6 and K4CBP6γ2 did not elicit toxic effects in *G. mellonella* larvae (**a**), nor did it induce haemolysis on sheep blood agar or in human erythrocytes (**b**, **c**). Additionally, neither compound reduced the viability of THP-1 monocytes (**d**), and rK4CBP6 exhibited no significant cytostatic activity against HT29 or HepG2 cell lines (**e**)

To investigate their potential harmful effects on animals, toxicity tests were conducted using a *G. mellonella* larvae model, which is widely recognized for its close similarity to mammalian innate immune responses, including those of humans [61, 62]. Larvae were treated with 200 µg ml^− 1^ of rK4CBP6 or K4CBP6γ2, and their survival rates were monitored. No significant differences were observed between treated larvae and untreated or IPS-treated groups, indicating that rK4CBP6 and K4CBP6γ2 do not exhibit acute toxicity in this model (Fig. 3a).

Certain plant defensins and peptide derivatives exhibit toxicity toward mammalian cells, which could limit their agricultural application [51, 63]. To address this concern, potential cell membrane-disrupting effects of rK4CBP6 and K4CBP6γ2 were evaluated using a hemolysis assay on sheep blood agar plates, 96-well plate-based assay on human erythrocytes, and colorimetric viability test employing THP1 human monocytes. At a high concentration of 200 µg ml^− 1^, neither rK4CBP6 nor K4CBP6γ2 induced hemolysis (Fig. 3b, c), or cell death of monocytes (Fig. 3d), confirming their lack of membrane disruption ability in mammalian cells.

To investigate the long-term cytostatic effects of rK4CBP6, we selected two model cells, a human colon-derived HT29 cell line and a liver-derived HepG2 cell line. HT29 cells are adherent epithelial cells commonly used in toxicology research owing to their characteristics of mature intestinal cells [64], while HepG2 cells serve as a widely used human hepatocyte model in drug toxicity, metabolism, and transport studies [65]. Compared to medium-treated control cells, cells treated with rK4CBP6 for over 24 h showed no significant decrease in cell viability and proliferative capacity, even after an extended incubation of 72 h (Fig. 3e).

These findings demonstrate that rK4CBP6 and K4CBP6γ2 exhibit no cytotoxicity in tested model systems. However, further research is necessary to evaluate their long-term in vivo effects, ecotoxicological impact, and potential interactions with mammalian immune systems to confirm their safety for widespread agricultural applications.

### Effect of rK4CBP6 and K4CBP6γ2 on Plant Seedlings

For the successful application of rK4CBP6 and K4CBP6γ2 as biofungicides, it is crucial that they do not negatively affect seedling growth or cause developmental delays in treated plants. The potential toxicity of rK4CBP6 and K4CBP6γ2 was investigated in seedlings of *M. truncatula* A-17 and *S. lycopersicum* L. cv. Ailsa Craig. *M. truncatula* is a widely used model organism, often employed to evaluate toxic effects of compounds in biological experiments [66]. *S. lycopersicum* also serves as another important model plant owing to its fleshy fruit, compound leaves, and relevance to the Solanaceae family, allowing insights to be extrapolated to related species [67]. Both species grow rapidly in vitro on water agar in Petri dishes, making them ideal for assessing toxicity of pesticide candidates [66, 67].

**Fig. 4:**
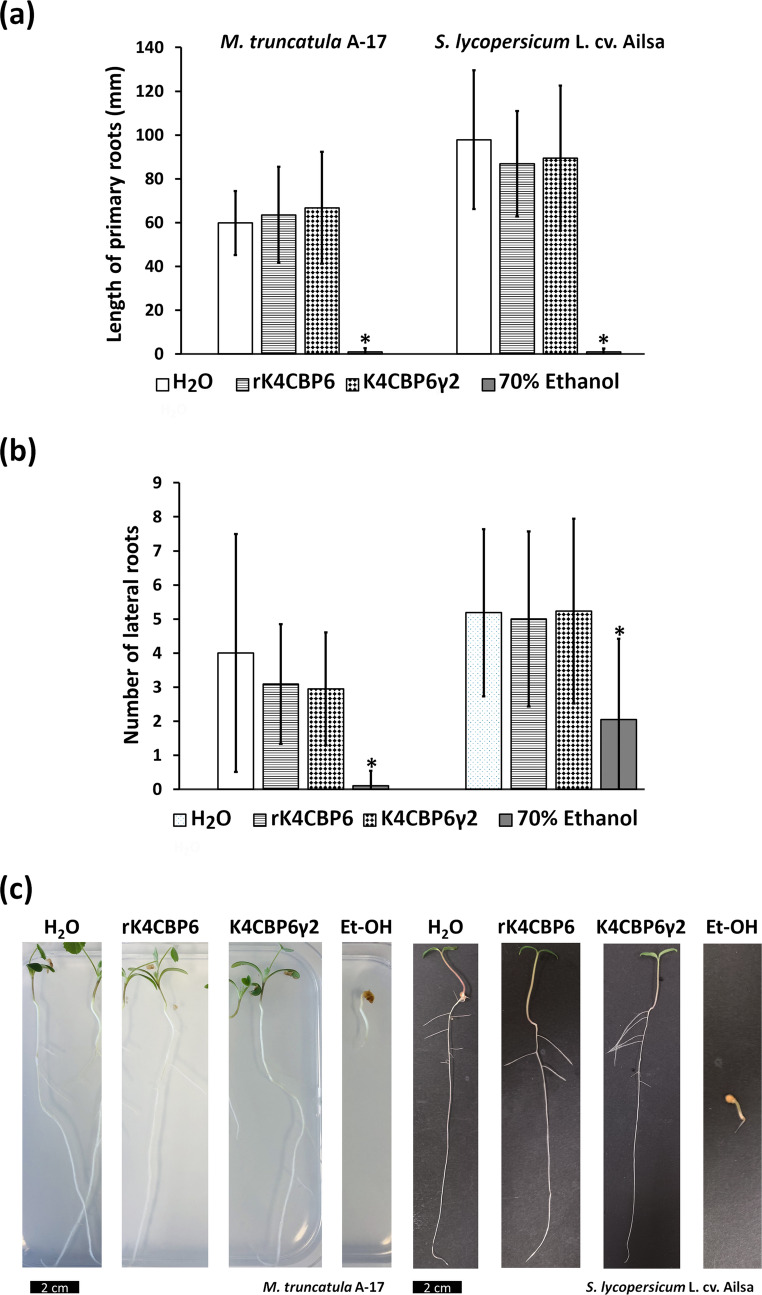
Root development and phenotype of *Medicago truncatula* A-17 and *Solanum lycopersicum* L. cv. Ailsa Craig after treatment with rK4CBP6 and K4CBP6γ2. (**a**) Primary root length and (**b**) lateral root number were assessed following treatment with rK4CBP6 and K4CBP6γ2 at 200 µg ml^− 1^ for 10 days (*M. truncatula*), and at 400 µg ml^− 1^ for 6 days (*S. lycopersicum*), respectively. Seedlings were maintained at 23 °C under photoperiodic day-night simulation (12/12 h light/dark cycle), with or without illumination at 1200 lx. (**c**) Phenotype of developed plants after the treatments. The results were compared to control groups treated with distilled water (H₂O) and 70% (v/v) ethanol. Bars represent the mean ± standard deviation of primary root length and lateral root number of seedlings (*n* = 20). Statistically significant differences were calculated using one-way ANOVA followed by Tukey’s HSD post-hoc test, comparing values against the untreated control. Significant differences were indicated with * (*p* ≤ 0.05; *n* = 20). Treatment with rK4CBP6 and K4CBP6γ2 did not lead to significant differences in primary root length (**a**) or lateral root number (**b**) when compared to each other. However, both treatments resulted in a reduction in primary root length relative to water-treated *S. lycopersicum* control plants

The effects of rK4CBP6 and K4CBP6γ2 were examined by treating *M. truncatula* and *S. lycopersicum* seedlings with 200 µg ml^− 1^ and 400 µg ml^− 1^ of defensin or peptide solution for 10 and 6 days, respectively. *M. truncatula* and tomato seedlings treated with rK4CBP6 and K4CBP6γ2. No significant differences were observed in primary root length (Fig. 4a) and number of lateral roots (Fig. 4b) between defensin/peptide-treated plants and water-treated control plants. *M. truncatula* seedlings developed into healthy mature plants after the treatments (Fig. 4c). In contrast, rK4CBP6 and K4CBP6γ2 moderately reduced root growth in *S. lycopersicum* seedlings (Fig. 4a).

These results suggest that rK4CBP6 and K4CBP6γ2 exhibit minimal toxicity to plant seedlings, supporting their potential as promising candidates for biofungicide applications. However, further studies are needed to explore their long-term effects on plant physiology to ensure safety and efficacy in agricultural settings.

### Proof-of-Concept Investigation - The Potential Applicability of rK4CBP6 and K4CBP6γ2 as Biofungicides

The antifungal susceptibility and toxicity tests confirmed that rK4CBP6 and K4CBP6γ2 fulfill key criteria for biofungicide candidates. To further evaluate their potential applicability in plant protection, experiments were conducted using established tomato plant–*B. cinerea* and *C. herbarum* model systems [48–50]. These two fungal species were selected based on our previous studies [48–50], in which their pathogenicity and virulence were thoroughly characterized using detached leaf and fruit inoculation assays. These results support their use as well-established plant-pathogen models in the present study.

**Fig. 5:**
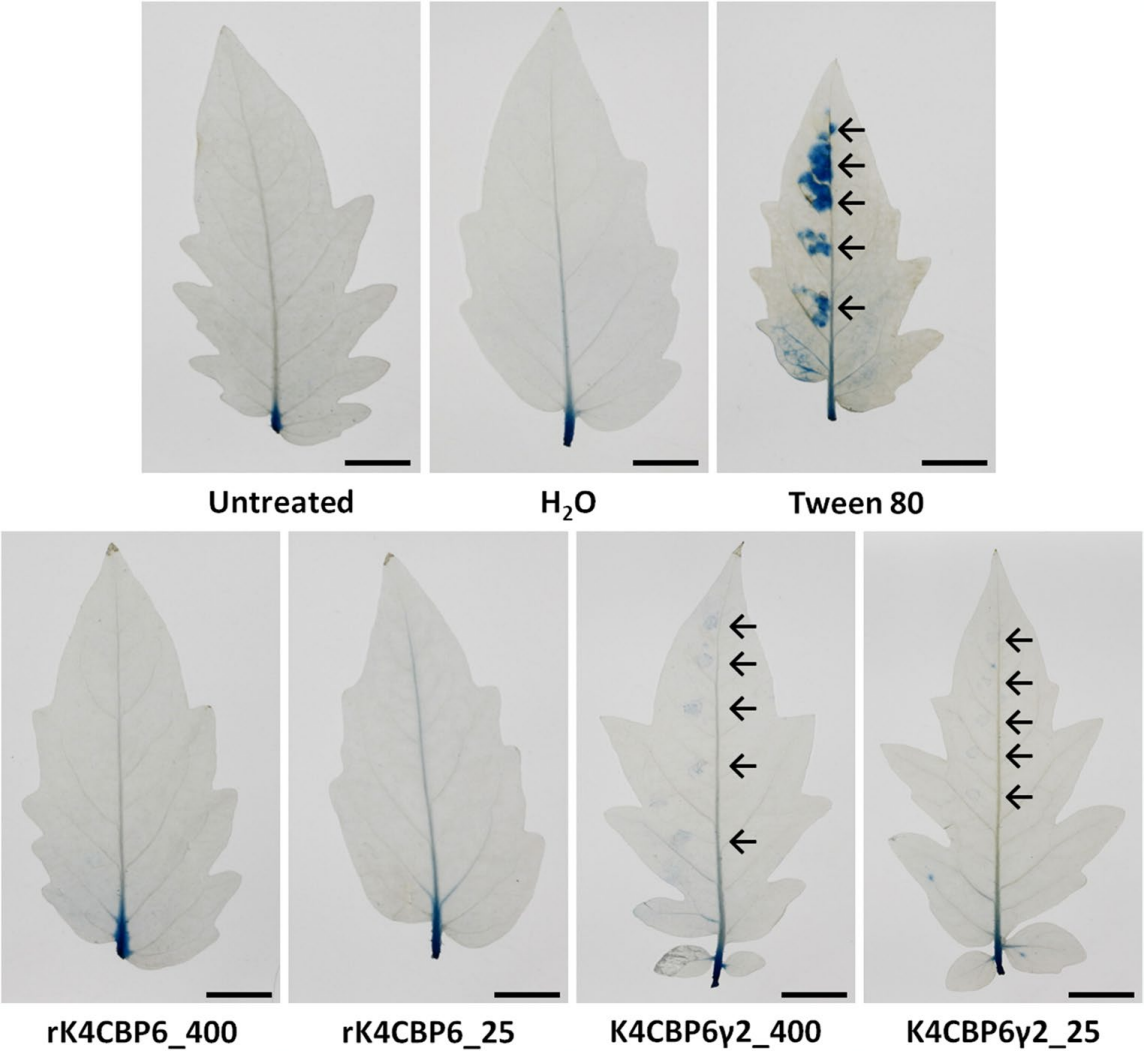
Evan’s blue staining of tomato plant leaves to assess the cytotoxic effects of rK4CBP6 and K4CBP6γ2. Leaves were treated with rK4CBP6 (400 µg ml^− 1^ and 25 µg ml^− 1^; rK4CBP6_400 and rK4CBP6_25, respectively) or K4CBP6γ2 (400 µg ml^− 1^ and 25 µg ml^− 1^; K4CBP6γ2_400 and K4CBP6γ2_25, respectively). The appearance of necrotic areas (blue-colored zones) in treated leaves was compared to control leaves, which were either left untreated (untreated) or treated with sterile water (H₂O) or 10% Tween80 (Tween80). Following treatment, the leaves were incubated at 23 °C, with 60% humidity, under a 12/12-h photoperiodic day-night cycle at 1200 lx for 4 days. The blue-coloured zones (marked with black arrows) on the leaves indicate cell death at the treatment points. Scale bars represent 1 cm. No visible tissue damage was observed in leaves treated with rK4CBP6, even at the highest concentration tested. In contrast, K4CBP6γ2 caused localized tissue damage at the sites of application

To assess their potential toxicity in plants, uninfected tomato leaves were treated with rK4CBP6 or K4CBP6γ2 at various concentrations, followed by Evan’s blue staining to visualize tissue damage [48]. rK4CBP6 did not disrupt leaf tissues, even at the highest concentration (Fig. 5). K4CBP6γ2, however, caused localized tissue damage at the treatment points, suggesting potential cytotoxic effects at certain concentrations (Fig. 5).

**Fig. 6:**
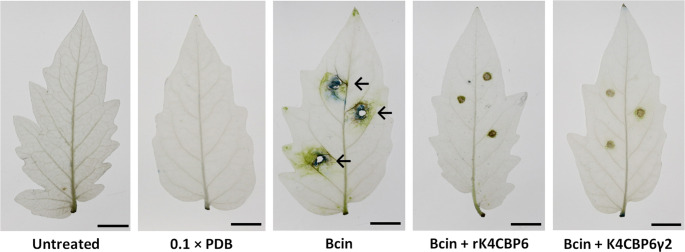
Evan’s blue staining of tomato plant leaves to assess the plant-protective potential of rK4CBP6 and K4CBP6γ2. Leaves were treated with 10 µl of 0.1 × PDB (control), 10 µl of *Botrytis cinerea* SZMC 21472 conidial suspension (1 × 10^7^ conidia ml^− 1^) (Bcin), 10 µl of *B. cinerea* SZMC 21472 conidial suspension (1 × 10^7^ conidia ml^− 1^) + rK4CBP6 (25 µg ml^–1^) (Bcin + rK4CBP6), 10 µl of *B. cinerea* SZMC 21472 conidial suspension (1 × 10^7^ conidia ml^− 1^) + K4CBP6γ2 (25 µg ml^− 1^) (Bcin + K4CBP6γ2). The blue-coloured zones (marked with black arrows) on the leaves indicate cell death at the treatment points. Scale bars represent 1 cm. Treatment with rK4CBP6 or K4CBP6γ2 conferred effective protection to the leaves, as evidenced by the absence of necrotic lesions and Evan's blue staining at the application sites. In contrast, extensive necrosis was observed in the untreated, infected control

The plant-protective potential of rK4CBP6 and K4CBP6γ2 was investigated using detached tomato leaves infected with *B. cinerea* conidia, a fungal pathogen responsible for substantial pre- and postharvest losses in over 200 plant species worldwide [29]. Untreated infected leaves exhibited extensive necrotic lesions with intense Evan’s blue staining. In contrast, the application of rK4CBP6 or K4CBP6γ2 at their MIC (25 µg ml^− 1^) successfully protected the leaves, with no necrotic lesions or intensive Evan’s blue staining were observed at the treatment sites (Fig. 6).

**Fig. 7:**
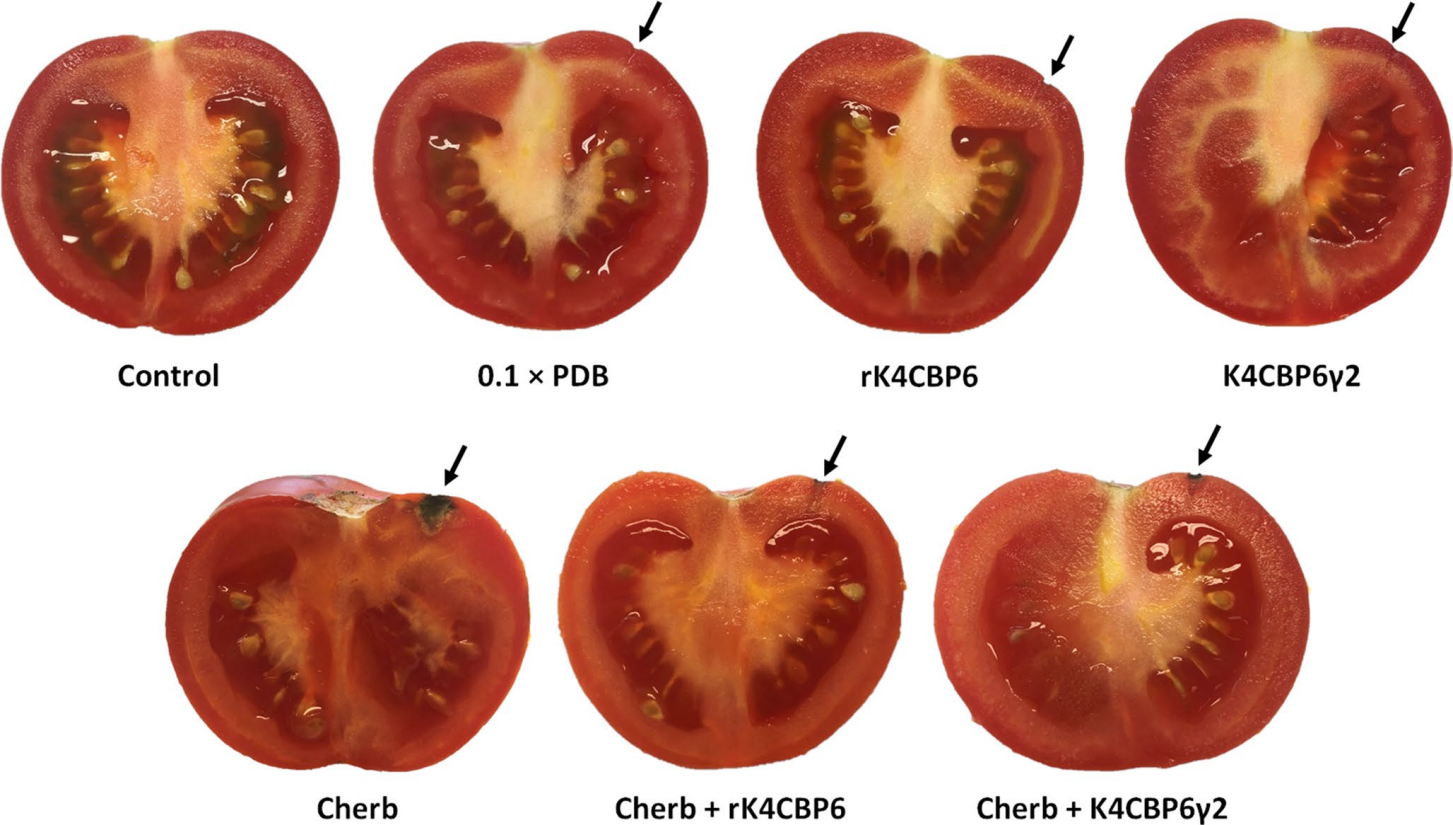
The fruit preservative potential of rK4CBP6 on postharvest tomato fruits infected with *Cladosporium herbarum* FSU 1148 after incubation at 23 °C for 7 days. The controls were uninfected but treated with medium (0.1 × PDB), rK4CBP6 (25 µg ml^− 1^), or K4CBP6γ2 (12.5 µg ml^− 1^). Tomato fruits infected with *C. herbarum* FSU 1148 (1 × 10^7^ conidia ml^− 1^) (Cherb) without defensin or peptide treatment served as infection control. Tomato fruits infected with *C. herbarum* FSU 1148 (1 × 10^7^ conidia ml^− 1^) were treated with rK4CBP6 (25 µg ml^− 1^) (Cherb + rK4CBP6) or K4CBP6γ2 (12.5 µg ml^− 1^) (Cherb + K4CBP6γ2). Unwounded tomato fruits without infection and treatment (Control) were used as natural decay controls. The sites of infections and treatments are indicated with black arrows. Treatment with rK4CBP6 or K4CBP6γ2 inhibited fungal infection and halted decay progression. In contrast, untreated infected tomato fruits exhibited pronounced fungal growth and tissue colonization, particularly at sting points and within deeper tissues

The efficacy of rK4CBP6 and K4CBP6γ2 in fruit protection was tested on tomato fruits infected with *C. herbarum*, a spoilage mold affecting fresh fruits and vegetables [68–70]. Control (0.1 × PDB) and defensin treatments (rK4CBP6 at 25 µg ml^− 1^, K4CBP6γ2 at 12.5 µg ml^− 1^) did not cause visible damage to fruit surfaces (Fig. 7). In untreated infected tomatoes, fungal growth and spread were observed at sting points and within deeper tissues (Fig. 7). Treatment with rK4CBP6 or K4CBP6γ2 at their MICs significantly inhibited fungal infection, preventing decay progression (Fig. 7).

Collectively, our findings confirm that rK4CBP6 and K4CBP6γ2 exhibit potent antifungal activity in planta. Their ability to suppress fungal infections in plants and fruits highlights their potential as effective contact fungicide candidates for plant disease management and postharvest crop protection.

## Discussion

In this study, we successfully generated functional rK4CBP6 in *K. phaffii*, demonstrating the feasibility of heterologous expression for production of this plant defensins. The achieved yield of 8 mg l^− 1^ is moderate compared to other defensins expressed in *K. phaffii*, with reported yields of 13.8 mg l^− 1^ for *Pisum sativum* defensin (Psd1) [56], 3 mg l^− 1^ for mung bean defensin (VrD1) [58], and over 60 mg l^− 1^ for Psd1 and *Venerupis philippinarum* defensin (VpDef) [57, 71]. Improper cleavage of extracellular signal sequences is a common issue in *K. phaffii-*based expression systems. This phenomenon has been reported in several cases, including Psd1 retaining four extra amino acids [56], VrD1 containing two additional amino acids [58], the maize defensin ZmD32 and PA1 knottin peptide, both exhibiting N-terminal sequence extensions [72, 73].

The widespread use of single-target-site chemical pesticides has contributed to the emergence of resistant fungal strains [74]. To mitigate this issue and effectively control fungal infections, multi-target solutions are preferable [75, 76]. Owing to their broad-spectrum antifungal activity and diverse modes of action, plant defensins have become a prominent focus in the search for effective biofungicides. Plant defensins can inhibit the growth of various economically significant plant pathogens in vitro [14, 58, 59, 77, 78]. Our results demonstrated that rK4CBP6 exhibits potent antifungal activity against *Botrytis*, *Cladosporium*, and *Fusarium* species, achieving complete growth inhibition at relatively low concentrations (Table 1). However, rK4CBP6 displayed limited or moderate antifungal activity against *Aspergillus* isolates. Notably, only a few plant defensins, such as MsDef1 and MtDef4, have demonstrated antifungal activity against *Aspergillus* species, inhibiting the growth of *Aspergillus flavus* [27].

Based on our observations, K4CBP6 can be classified as a non-morphogenic plant defensin, as it did not induce visible hyphal hyperbranching or other morphogenetic changes in the tested fungal strains. This sets it apart from morphogenic defensins, such as RsAFP2 and MsDef1, which typically elicit extensive alterations in fungal growth patterns by interacting with cell wall components and activating signalling pathways related to septin organization and cell wall integrity [79, 80]. In contrast, non-morphogenic defensins, including NaD1, NsD7, and HsAFP1, exert their antifungal activity primarily through direct interactions with specific membrane lipids, resulting in rapid membrane permeabilization, ion leakage, and subsequent cell death [81, 82]. The γ-core motif has been identified as a key determinant of both antifungal potency and morphogenic behavior; for example, amino acid substitutions in this region can convert morphogenic defensins into non-morphogenic ones and vice versa [27]. In this context, the activity profile of K4CBP6, marked by the absence of morphogenetic effects and its rapid fungicidal action, strongly supports its classification as a non-morphogenic defensin. Future studies, such as lipid-binding assays and targeted γ-core mutagenesis, may provide further insights into the molecular basis of its antifungal mechanism.

Sagaram et al. [27] investigated synthetic peptides spanning the γ-core motif of *Medicago truncatula* defensin (MtDef4) and *Medicago sativa* defensin (MsDef1). Their findings revealed that the amino acid composition of the γ-core motif determines their antifungal mechanism. An abundant presence of hydrophilic, positively charged amino acids induces fungal cell death, while a higher proportion of hydrophobic and negatively charged amino acids promotes morphogenetic changes in hyphae. Consequently, the γ-core motif of MtDef4 inhibited the growth of *F. graminearum*, whereas the γ-core peptide derivatives of MsDef1 were ineffective against this fungal pathogen [27]. Similar investigations have been conducted on several plant defensins, including radish seed defensin (Rs-AFP2), *M. truncatula* defensin (MtDef5), and *P. vulgaris* seed defensin (PvD1) [60, 83–86]. Our investigations with K4CBP6γ1 and K4CBP6γ2 highlighted the importance of positive charge and hydrophobicity in antifungal activity. K4CBP6γ2, which is positively charged and highly hydrophilic, inhibited fungal growth at similar concentrations to rK4CBP6 (Tables 1 and 2). Notably, it exhibited complete inhibition of *F. subglutinans* at 25 µg ml^–1^, whereas the full-length protein did not (Table 1). K4CBP6γ1, which is neutral and slightly hydrophilic, was ineffective against most of the tested fungal isolates (Table 1), suggesting that positive amino acid residues are essential for antifungal efficacy. However, as Sagaram et al. [27] demonstrated, charge alone is not always sufficient to confer antifungal activity.

We proved that neither rK4CBP6 nor K4CBP6γ2 affected the survival of *G. mellonella* larvae, did not exhibit membrane-disrupting effect on human erythrocytes, or caused cell death of THP1 human monocytes, furthermore they did not decrease the cell viability and proliferation capacity of HT29 and HepG2 cells (Fig. 3). These findings align with those of Skalska et al. [87, 88], which demonstrated that *P. vulgaris* defensin (PvD1) does not disrupt human red blood cell membranes or cause toxicity in breast epithelial cells (MCF 10 A). In line with our findings, SolyC peptide (spanning the sequence of K4CBP6γ2) has shown to exhibit < 5% hemolytic activity at 50 µg ml^− 1^ concentration and no toxicity toward THP1 cells at 60–120 µg ml^− 1^ concentration [51].

rK4CBP6- and K4CBP6γ2-treated *M. truncatula* and *S. lycopersicum* seedlings developed into healthy plant, although the length of primary root was decreased in *S. lycopersicum* seedlings (Fig. 4). A few studies have shown negative effects of plant defensins on the growth and development of plants. Allen et al. [89] demonstrated that defensins from *M. sativa* (MsDef1), *M. truncatula* (MtDef2), and *R. sativus* (RsAFP2) inhibit root growth of *Arabidopsis thaliana* seedlings. However, this inhibition was not observed in *M. truncatula* [89]. Consistent with findings of Allen et al. [89], root growth of *M. truncatula* remained unaffected when treated with 200 µg ml^− 1^ of rK4CBP6 or K4CBP6γ2 (Fig. 4), which is eight times higher than the MIC determined against *B. cinerea*. However, compared to published data, their negative effects on primary root growth were negligible, even at the high concentration (400 µg ml^− 1^) [89].

Few studies have reported that plant defensins or their γ-core peptide derivatives can directly protect plants against phytopathogenic filamentous fungi. Velivelli et al. [90] showed that NCR044, a nodule-specific cysteine-rich peptide from *M. truncatula*, reduces *B. cinerea* infection in lettuce leaves, rose petals, *Nicotiana benthamiana*, and tomato plants. Tetorya et al. [16] demonstrated that wild-type and modified γ-core peptides of MtDef4 suppress *B. cinerea* infection in *N. benthamiana* leaves. Like NCR044 and MtDef4 defensins, rK4CBP6 and K4CBP6γ2 retained their antifungal activity and suppressed *B. cinerea* infection, confirming their potential role in plant protection.

In summary, our study demonstrates that the recombinant K4CBP6, produced via a heterologous expression system, and its chemically synthesized γ-core peptide derivative, exhibit potent antifungal activity in planta under laboratory conditions, without inducing significant toxicity in plant or animal models. These findings support their potential application as promising candidates for the development of plant-derived biofungicides. Future research should focus on assessing their environmental stability, formulation strategies, and long-term ecotoxicological effects. In addition, field trials will be essential to determine their suitability for large-scale agricultural applications.

## Supplementary Information

Below is the link to the electronic supplementary material.

Supplementary Material 1

## Data Availability

The authors declare that all data supporting the findings of this study are available within the article and its supplementary information files are available from the corresponding author upon request.
