# Supplementary material for: Characterization and Biofungicide Potential of a Novel Antifungal defensin, K4CBP6, from Solanum lycopersicum L
Source: Probiotics Antimicrob Proteins. 2025 Dec 8;18(4):6018–37. doi: 10.1007/s12602-025-10865-z (PMC13341736; doi:10.1007/s12602-025-10865-z)
Supplement: Supplementary file 1 — Supplementary Material 1 [file 12602_2025_10865_MOESM1_ESM.docx]

**Supplementary Information**

Journal name: Probiotics and Antimicrobial Proteins

**Title: Characterization and biofungicide potential of a novel antifungal defensin, K4CBP6, from *Solanum lycopersicum* L.**

**Rebeka Papp^1,2^, Péter Poór^3^, Zalán Czékus^3^, Györgyi Váradi^4^, Zoltán Kele^4^, Attila Borics^5^, Gábor Bende^1,2,6^, Kata Horváti^7^, Gábor K. Tóth^4,8^, László Galgóczy^1*^, Liliána Tóth^1*^**

^1^Department of Biotechnology and Microbiology, Faculty of Science and Informatics, University of Szeged, Szeged, Hungary;

^2^Doctoral School of Biology, Faculty of Science and Informatics, University of Szeged, Szeged, Hungary;

^3^Department of Plant Biology, Faculty of Science and Informatics, University of Szeged, Szeged, Hungary; ^4^Department of Medical Chemistry, Albert Szent-Györgyi Medical School, University of Szeged, Szeged, Hungary;

^5^Institute of Biochemistry, HUN-REN Biological Research Centre, Szeged, Hungary;

^6^Department of Theoretical Health Sciences and Health Management, Faculty of Health Sciences and Social Studies, University of Szeged, Szeged, Hungary;

^7^Institute of Materials and Environmental Chemistry, HUN-REN Research Centre for Natural Sciences, Budapest, Hungary;

^8^MTA-SZTE Biomimetic Systems Research Group, University of Szeged, Szeged, Hungary

^*^ Correspondence: László Galgóczy; galgoczi@bio.u-szeged.hu

| **Table S1. Equipment used in the experiments (names, manufacturers, and technical specifications)** | | | | |
| --- | --- | --- | --- | --- |
| **EQUIPMENT** | **MANUFACTURING COMPANY** | **TASK** | **PROPERTIES** |  |
| Bio-Scale™ Mini Macro-Prep High S column | Bio-Rad Laboratories, Hercules, CA, USA | Purification of protein from the cell-free supernatant | Strong cation exchanger column |  |
| NGC Medium-Pressure Liquid Chromatography instrument | Bio-Rad Laboratories, Hercules, CA, USA |  |  |  |
| SnakeSkin™ dialysis tubing | Thermo Fisher Scientific, Waltham, MA, USA | Dialyzation of the pure protein fractions | 3.5 K MWCO |  |
| Orbitrap mass spectrometer (Thermo Q-Exactive+) | Thermo Fisher Scientific, Waltham, MA, USA | Determination of the molar mass of protein |  |  |
| Nano Equity Ultraperformance Liquid Chromatography System | Waters, Milford, MA, USA |  | Ultra-high-performance liquid chromatography system |  |
| Phenomenex Jupiter C18 column | Phenomenex, Torrance, CA, USA | Purification of the protein | 250 × 10 mm, 10 μm particle size, 300 Å pore size |  |
| Agilent-Shimadzu apparatus | Agilent Technologies, Santa Clara, CA, USA |  |  |  |
| Phenomenex Jupiter C18 column | Phenomenex, Torrance, CA, USA | Analyzation of the protein | 250 × 10 mm, 10 μm particle size, 300 Å pore size |  |
| Agilent 1100 Series liquid chromatograph | Agilent Technologies, Santa Clara, CA, USA |  |  |  |
| Jasco-J815 electronic circular dichroism spectrometer | JASCO, Tokyo, Japan | Investigtion of the secondary structure and thermal stability of the protein | The far-UV range (185–260 nm) |  |
| Peltier thermoelectric controller | TE Technology, Traverse City, MI, USA |  | We used this to set the temperature |  |
| Phenomenex Jupiter C18 column | Phenomenex, Torrance, CA, USA | Purification of the peptides | 250 × 10 mm, 10 μm particle size, 90 Å pore size |  |
| Phenomenex Luna C18 column | Phenomenex, Torrance, CA, USA | Analyzation of the peptides | 250 × 4.6 mm, 10 μm particle size, 100 Å pore size |  |
| Microplate Reader 96 & 384 Thermo Multiskan Ascent | Thermo Fisher Scientific, Waltham, MA, USA | *In vitro* antifungal susceptibility test |  |  |
| Thermo Multiscan GO plate reader | Thermo Fisher Scientific, Waltham, MA, USA | Hemolysis assay |  |  |

**Table S2 Close K4CBP6 homologs in the kingdom Plantae detected by a BLAST search in the UniProt database [1]**

| **Protein** | **Origin** | **ID** | **Homology** |
| --- | --- | --- | --- |
| Knottin scorpion toxin-like domain-containing protein | *Solanum lycopersicum* | A0A3Q7H3Y0 | 100% |
| Knottin scorpion toxin-like domain-containing protein | *Solanum commersonii* | A0A9J5Y4T1 | 92% |
| Knottin scorpion toxin-like domain-containing protein | *Solanum verrucosum* | A0AAF0TXB4 | 88% |
| Defensin P322 | *Solanum tuberosum* | K7WJX9 | 87% |
| Defensin P322 | *S. tuberosum* | M1B7U1 | 85% |
| Knottin scorpion toxin-like domain-containing protein | *Anisodus tanguticus* | A0AAE1SHN7 | 82% |
| Defensin protein | *S. lycopersicum* | B1N678 | 82% |
| Knottin scorpion toxin-like domain-containing protein | *S. verrucosum* | A0AAF0TXS8 | 81% |
| Knottin scorpion toxin-like domain-containing protein | *Solanum commersonii* | A0A9J5Y1K1 | 81% |
| Knottin scorpion toxin-like domain-containing protein | *Anisodus acutangulus* | A0A9Q1RLI9 | 81% |
| Defensin-like protein | *Capsicum annuum* | A0A2G2Z0J1 | 81% |
| Defensin-like protein | *Capsicum baccatum* | A0A2G2WB60 | 81% |
| Defensin | *C. annuum* | Q8W4V6 | 79% |
| Defensin protein | *S. tuberosum* | M1B7U0 | 79% |
| Defensin-like protein P322 | *Nicotiana sylvestris* | A0A1U7W5D8 | 77% |
| Defensin-like protein P322 | *Nicotiana tabacum* | A0A1S4BPM7 | 77% |
| Defensin-like protein P322 | *N. sylvestris* | A0A1U7VU16 | 76% |
| DEF1 protein | *N. tabacum* | A0A0C4FST6 | 76% |
| Defensin-like protein | *C. baccatum* | A0A2G2WBB6 | 76% |
| Defensin-like protein P322 | *N.tabacum* | A0A1S3YG04 | 74% |
| Knottin scorpion toxin-like domain-containing protein | *S. commersonii* | A0A9J5Y4B2 | 74% |
| Knottin scorpion toxin-like domain-containing protein | *Vigna angularis* var. *angularis* | A0A0S3R3M0 | 74% |
| Defensin-like protein P322 | *N. tabacum* | Q9MB66 | 74% |
| Knottin scorpion toxin-like domain-containing protein | *S. verrucosum* | A0AAF0R973 | 73% |
| Defensin-like protein P322 | *N. sylvestris* | A0A1U7XZ26 | 73% |
| Defensin-like protein P322 | *N. tabacum* | A0A1S4D6A4 | 73% |
| Defensin D2 | *Phaseolus vulgaris* | F8QXQ0 | 72% |
| Knottin scorpion toxin-like domain-containing protein | *Anisodus tanguticus* | A0AAE1SH56 | 72% |
| Defensin Ec-AMP-D2 | *V. radiata* var. *radiata* | A0A1S3UP76 | 71% |
| Defensin-like protein | *Cajanus cajan* | A0A151SD74 | 71% |
| Knottin scorpion toxin-like domain-containing protein | *Sphenostylis stenocarpa* | A0AA86S512 | 71% |

pEX-A128-K4CBP6new_Pichia plasmid

**Kex2 signal cleavage**

**Ste13 signal cleavage**

***Xho*I signal cleavage**

***GGCCCTCGAGAAAAGAGAGGCTGAAGCT***AGGCATTGTGAAAGTTTGTCCCACCGTTTCAAGGGTCCATGCGTCAGTGATAAGAACTGTGCTTCTGTCTGTGAAACAGAAAGATTCTCTGGAGGTAACTGTCGTGGCTTTCGTAGAAGGTGCTTCTGTACC***AAGCCATGCTAATCTAGAGGCC***

***Xba*I signal cleavage**

**Fig. S1.** │ Nucleotide sequence of the cDNA encoding the K4CBP6 protein inserted into the pEX-A128-K4CBP6new_Pichia plasmid. The *Xho*I restriction site, the Kex2 signal sequence, and the Ste13 signal sequence are highlighted in yellow. Glu-Ala repeat is framed in black. The cDNA encoding the K4CBP6 defensin is shown in blue, while the *Xba*I restriction site is indicated in green. The cleavage sites are marked with arrows. The binding sites of the primer pair (K4CBP6F: 5′-GGC CCT CGA GAA AAG AGA GGC TGA AGC T-3′ and K4CBP6R: 5′-GGC CTC TAG ATT AGC ATG GCT T-3′) used for amplification are indicated in bold italic letters


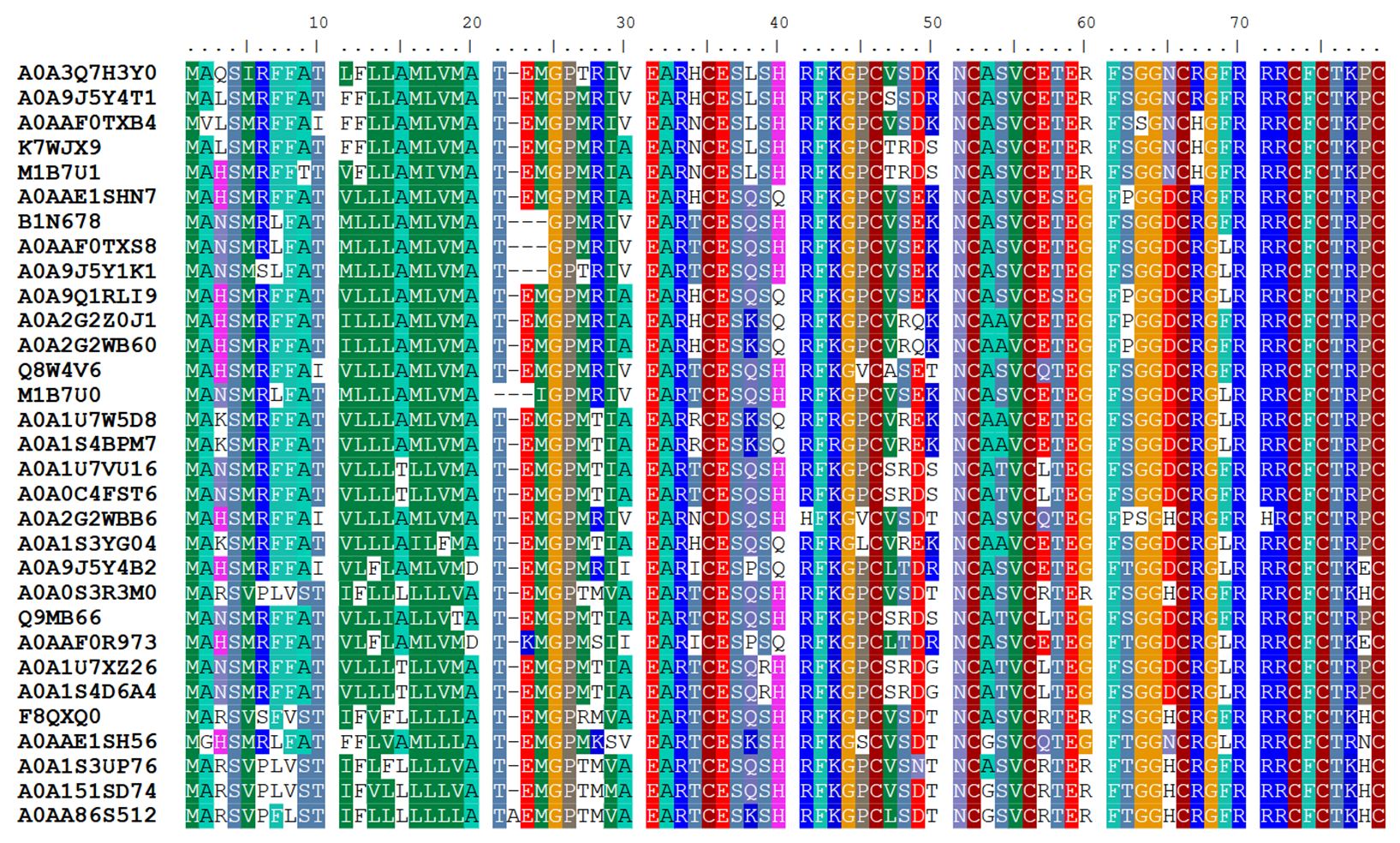


**Fig. S2.** │ The amino acid sequence alignment of K4CBP6 homologs identified in the kingdom Plantae. Homologs (with maximum 71% identity in primary structure) were detected through BLAST searches conducted on published plant genomes available in the UniProt database [1]. The y-core regions are marked with black frames **Table S2** provides the UniProt identification numbers and origin corresponding to the detected homologs

**REFERENCE**

**[1]** Bateman A, Martin MJ, Orchard S, Magrane M, Adesina A, Ahmad S, Bowler-Barnett EH, Bye-A-Jee H. Carpentier D, Denny P, Fan J (2025) UniProt: the universal protein knowledgebase in 2025. Nucleic Acids Res 53:D609–D617. https://doi.org/10.1093/nar/gkae1010
